# Resentment, Hate, and Hope in Amyotrophic Lateral Sclerosis

**DOI:** 10.3389/fpsyg.2012.00530

**Published:** 2012-11-27

**Authors:** C. Oster, F. Pagnini

**Affiliations:** ^1^The Healers CampaignHaslett, MI, USA; ^2^Department of Psychology, Catholic University of MilanMilan, Italy; ^3^Niguarda Ca’ Granda HospitalMilan, Italy

**Keywords:** anger, resentment, hope, amyotrophic lateral sclerosis, quality of life, caregivers, ALS, will to live

## Abstract

Amyotrophic lateral sclerosis (ALS) is a fatal and progressive neurodegenerative disease. Despite much research having been conducted about psychological issues involved in living with ALS, anger, and resentment have yet to be investigated. Moreover, the construct of “hope” has received little attention, so far. An online survey was created to investigate hate, resentment, and hope issues in people with ALS, in relation to the willingness to adopt a strict nutrient-dense diet if it were shown to increase longevity. Results indicate that there is a high level of hope in the sample. People who have lived with ALS for more time expressed a higher level of hope to live 10 years or more. Those who are married were more likely to have hope of living 10 years or longer and more likely to have lower levels of hate against ALS. Dietary self-care choices appear to be related to hope issues. Resentment and hate tended to be higher in people who have had ALS for less time, and in women. Despite some methodological limitations, the results suggest that hope, hate, and resentment could be important issues to explore in future studies.

## Introduction

Amyotrophic lateral sclerosis (ALS) is a fatal and progressive neurodegenerative disease, which affects motor neurons in the anterior horn of the spinal cord, the brainstem, and the motor cortex. It is a rare disease, with an incidence of 1.89 per 100,000/year in western countries (Worms, 2001). People with ALS experience a progressive muscle weakness, becoming relentlessly immobile, developing impaired speech, and respiration problems. In the late stages of the disease, progressing paralysis can result in a “locked-in” state in which only residual muscular movement is possible.

Despite the average life expectancy of about 3 years after symptom onset (Ilzecka et al., 2003), the progression of the disease is unpredictable, with 10% of patients with ALS living more than 10 years (Andrews, 2009). So far, research has not demonstrated that it is possible to stop the progression of the disease. Therefore, the improvement of patients’ quality of life is often considered one of the primary goals of clinical management (Simmons, 2005; Pagnini, 2012).

Many psychological issues have been investigated in ALS, including psychological well-being, anxiety, and depression (Pagnini et al., 2012a), pain (Brettschneider et al., 2008), hopelessness (Plahuta et al., 2002), spiritual and existential well-being (Dal Bello-Haas et al., 2000). However, no study to our knowledge has investigated the topics of hate and resentment. Moreover, despite its clinical importance (Fanos et al., 2008), the issue of hope has received little empirical investigation. To date, no study has investigated the relationship among hate, resentment, and hope and their development through time.

Hate and resentment, as well as hope, are often reported as a psychological response toward a chronic and non-treatable illness. Clinical practice (Radunovic et al., 2007), as well as patients’ perspectives (Melazzini, 2007), often report of anger reactions, expressed in various ways by the patient. Not surprisingly, a study by Palmieri et al. (2010) observed high levels of anger in the considered ALS population. The “relationship” established with the disease seems to change over time (De Groot et al., 2007), probably because of the advancement of the disease and/or a grief elaboration process. Clinical practice suggests that ALS is often the target of hate by the people who have it; resentment is another emotion expressed by the patients, as well as by their caregivers, but there are very few scientific data pertaining to those reactions.

The diagnosis of ALS may threaten hope, especially with the belief that there is nothing that can be done to change a patient’s fate. However, many clinicians suggest that “hope” is a very important issue for the psychological well-being of patients and their caregivers (Centers, 2001). In the ALS scientific literature, the concept of “hope” is less investigated than the one of “hopelessness” (Plahuta et al., 2002). Hopelessness is related to depressive features, and with low levels of quality of life (Ganzini et al., 1999); moreover, hopelessness seems to predict the desire for assisted suicide (Ganzini et al., 2002).

Our rationale in this study was to investigate the prevalence and degree of hate and resentment in a sample of the ALS population, and to understand their relationships with time since diagnosis, hope, marital status, and other demographic characteristics. A further innovation of this research is the examination of these three variables in relationship to the willingness to extend one’s longevity through nutritional self-care. This variable was included, because clinical considerations made by Oster (the manuscript’s first author, who is an 18 years ALS survivor) suggest that some people with ALS would not be willing to adopt a healthier diet even if it were to be shown to extend life. We wanted to investigate the prevalence of this mindset in our study’s subject population, and to discover whether the variables of hate, resentment, and hope were related to this attitude.

## Materials and Methods

An online questionnaire was created with a Google spreadsheet, as part of a larger study. The overall survey was composed by 65 items, with both opened-ended and closed-ended questions; in case of closed-ended questions, subjects could choose among different sentences. The time requested to complete the entire survey ranged between 10 and 20 min. The survey was created with Google Documents tool and was accessible by subjects through a dedicated template. All the items were generated *ex novo* (therefore no cut-off-points could be expected). There were no forced answers. For the purpose of this study, we only used a selected sub-sample of items.

People with ALS were invited with messages that were posted in ALS forums, facebook, and a newsletter, as well as with an announcement on “The Healers” website. The purpose of the survey was described before subjects were asked to complete the questionnaire, stating that results would be analyzed only for research purposes, and there was no possibility to link the published data to their true identity.

Together with demographical data, subjects were asked to provide answers about hate, resentment, and hope, with questions like “Do you believe that there will be a cure for ALS in your lifetime?” and “What statement best describes the level of resentment that you feel about living with ALS?” Multiple answers were possible.

The analyses, both descriptive and inferential, were, carried out with Excel and SPSS software, are mainly descriptive statistics and cross-tabs analysis, using the Pearson’s chi-squared test.

## Results

A total of 83 subjects with ALS completed the survey. The sample’s characteristics are reported in Table 1. Missing values were excluded.

**Table 1:** **Sample characteristics (*N* = 83)**.

		Frequency	%
Months since diagnosis [*M*, (SD)]	44.12 (84.32)		
Sex	Female	41	50
	Male	41	50
Marital status	Now married	56	68.3
	Widowed	4	4.9
	Divorced	11	13.4
	Separated	3	3.7
	Never married	3	3.7
	other	5	6.1
Highest degree	Less than high school	1	1.2
	High school graduate	18	22.2
	Some college credit	15	18.5
	Associate degree (e.g., AA, AS)	5	6.2
	Bachelor’s degree (e.g., BA, AB, BS)	23	28.4
	Master’s degree (e.g., MA, MS, MEng, MEd, MSW, MBA)	12	14.8
	Professional degree (e.g., MD, DDS, DVM, LLB, JD)	4	4.9
	Doctorate degree (e.g., PhD, EdD)	2	2.5
	other	1	1.2
Work activity	Employed for wages	13	15.9
	Self-employed	4	4.9
	Unemployed	12	14.6
	Retired	39	47.6
	Unable to work	13	15.9
	Other	1	1.2
Race	Asian	1	1.2
	Black or African American	1	1.2
	White	76	92.7
	Hispanic	4	4.9
Country/province	USA	73	89
	South America	1	1.2
	Europe	5	6.1
	Australia	1	1.2
	Canada	1	1.2
	Mexico	1	1.2
Life environment	City	28	34.1
	Country/Rural	10	12.2
	Suburban	28	34.1
	Small town	15	18.3
	Other	1	1.2

**Figure 1:**
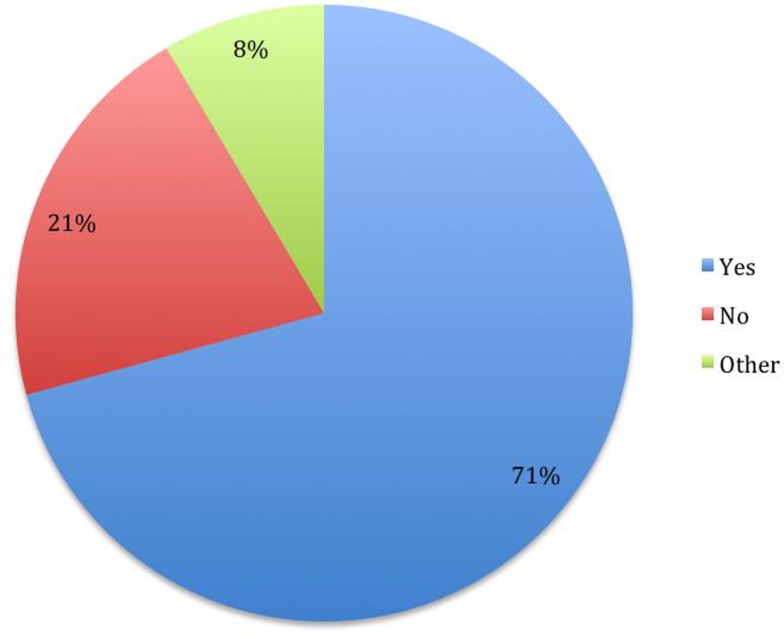
**Do you have hope that you could live 10 years or longer regardless of whether an effective pharmaceutical treatment were to be discovered? (*N* = 82)**.

**Figure 2:**
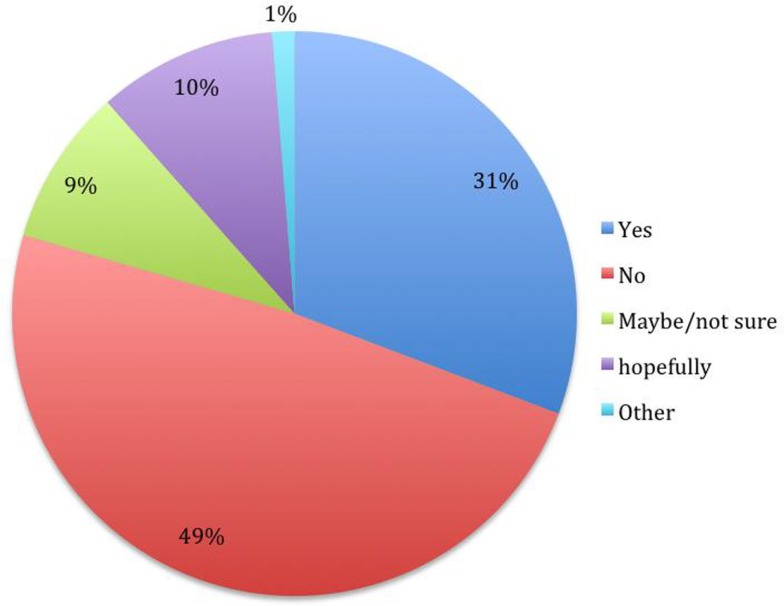
**Do you believe that there will be a cure for ALS in your lifetime? (*N* = 78)**.

**Figure 3:**
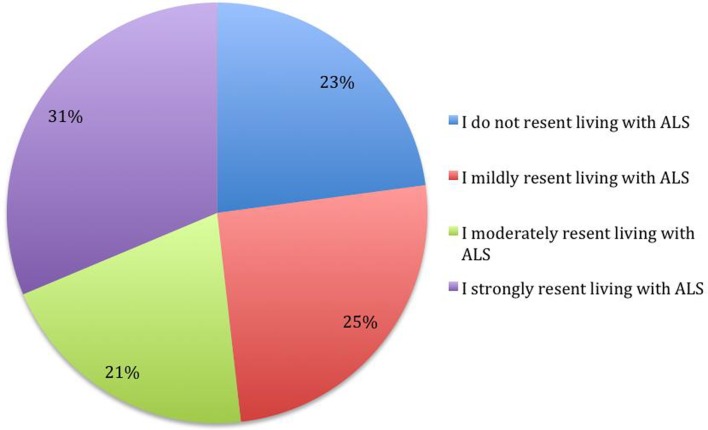
**What statement best describes the level of resentment that you feel about living with ALS? (*N* = 83)**.

**Figure 4:**
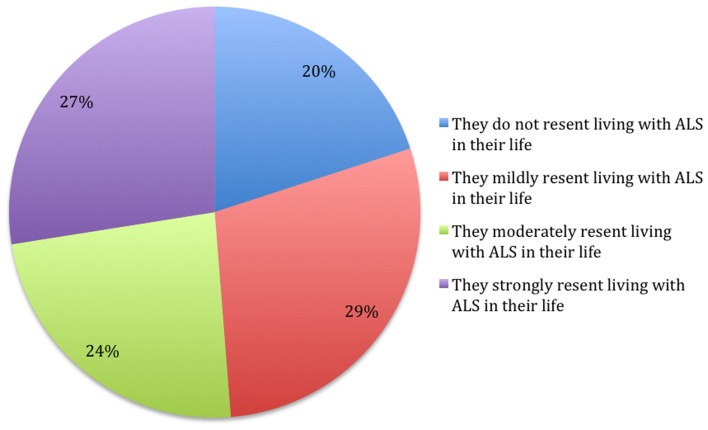
**What statement do you guess best describes the level of resentment that your primary caregiver feels about living with ALS in their life? (*N* = 80)**.

**Figure 5:**
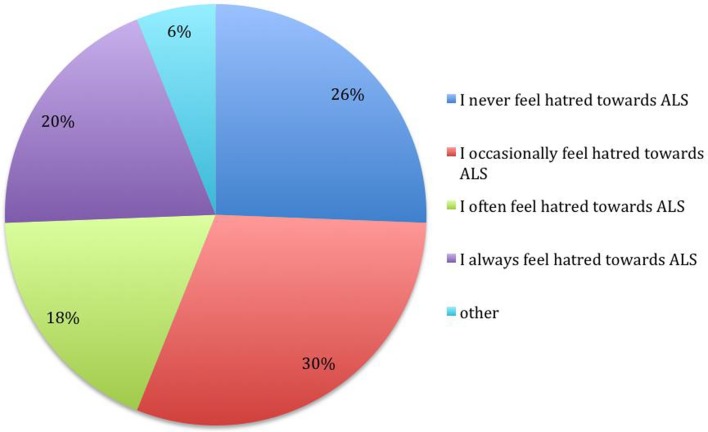
**What statement most accurately describes your feelings in the last 6 months regarding “hating” ALS? (*N* = 82)**.

The answers provided to the questions are presented in the Figures 1–5.

People who answered positively to the question about the hope to live more than 10 years had a lower frequency of resentment, χ^2^ (6, *N* = 82) = 11.14, *p* < 0.05, and hate χ^2^ (6, *N* = 81) = 13.48, *p* < 0.05, toward the illness.

The level of resentment of ALS reported by subjects is highly related to the level of resentment perceived in the caregiver, χ^2^ (6, *N* = 81) = 41.09, *p* < 0.001, and the level of hate toward ALS, χ^2^ (6, *N* = 81) = 58.21, *p* < 0.001. The caregiver’s resentment perceived by the patient is also related to the level of hate expressed by the subject, χ^2^ (6, *N* = 80) = 21.19, *p* < 0.05. It seems that there is a tendency for a positive correlation among these three variables.

Subjects who have the hope to live 10 or more years would be more willing to follow a strict diet if it would increase the life expectancy by 1 year χ^2^ (6, *N* = 75) = 21.93, *p* < 0.01, but no differences were found about the willingness to take a new drug with uncomfortable side effects. Both the questions about “hope” presented no differences with regards to the demographic variables.

Women report higher level of resentment, with higher frequencies of moderate/strong resentment answers, in comparison with males χ^2^ (3, *N* = 81) = 6.44, *p* < 0.05. There is a similar tendency, non-significant (*p* < 0.1), with women reporting a higher hate level. Splitting the sample into two groups, using the median of the time since diagnosis, subjects who have had the disease for less time present with higher level of resentment χ^2^ (3, *N* = 81) = 8.05, *p* < 0.05, and hate χ^2^ (3, *N* = 82) = 9.89, *p* < 0.05.

The subjects who report a higher level of caregiver’s resentment indicate with greater frequency the answer “yes” to the question “Do you believe that there is a relation between your dietary habits and the losses that you have experienced subsequent to ALS?” χ^2^ (9, *N* = 78) = 20.32, *p* < 0.05.

Regarding hope, people who do not believe that there will be a cure for ALS in their lifetime were more likely to believe that they will not live for 10 years, compared to those who have the hope that a cure will be found χ^2^ (1, *N* = 78) = 4.67, *p* < 0.05. People who answered “yes” to both the hope questions (*N* = 21), compared to those who answered “no” (*N* = 11), had ALS for a longer time (but without statistical differences), were more likely to be married χ^2^ (5, *N* = 32) = 13.38, *p* < 0.05 and reported a lower level of hate against ALS χ^2^ (4, *N* = 32) = 8.06, *p* < 0.05.

Considering the resentment level expressed by the subjects, in comparison with the resentment level that the subjects reported to be experienced by the caregivers, about half of the subjects (51.2%) express an equal level of resentment, the 21.3% report higher level of resentment experienced by the patient, while the 27.5% indicate the contrary. No differences arose about demographics or hope levels reported (all Chi Squares without significant results).

## Discussion

The present study from an online survey completed by men and women with ALS and analyzed with cross-sectional analyses – aimed to investigate levels of hate, resentment, and hope, and the relationships among them. These variables were also examined in relationship to the willingness to extend longevity through a commitment to disciplined nutritional self-care. To our knowledge, this is one of the first attempts to investigate, with a survey methodology, the point of view of people with ALS about the hope for longevity and a cure, and the level of hate and resentment toward the illness. Furthermore, we are not aware of any study examining motivation regarding nutritional self-care, related to the will to live.

In our sample, most of the subjects (70%) hoped to be able to live more than 10 years. The hope for a cure was somewhat less present. A third of the people who reported hope of seeing a cure in their lifetime, and more than half of the sample who reported not believing that they will have the chance to see a cure. These two issues were correlated. Interestingly, people in a marriage were more likely to have hope of living 10 years or longer and more likely to have lower levels of hate against ALS.

To hate ALS involves hating having ALS and all of the potentially frustrating and otherwise emotionally challenging experiences that occur in the lives of people with ALS, as well as hating the way one’s own physiology is performing. Perhaps, our finding regarding marriage and hope suggests that people in marriages may build a vision of hope together, and that the meaning of their shared marital commitment could be protective against what is involved in hating one’s life with ALS. Research into this area could give clinicians greater understanding of strategies that could facilitate greater hope as well as less resentment and hate in people with ALS. It must be pointed out that some people with ALS may value hating and resenting ALS as the best strategy for them in terms of coping and life satisfaction, and we have much to learn from them about their experiences in living with ALS.

The majority of the sample indicated a strong or moderate resentment level; a similar indication was given subjects’ perception of caregiver resentment level. About a third of the sample reported sporadic episodes of hate, while less than a fifth of the subjects indicated to always hate the disease. The current study suggests a prevalence of these distressing emotional experiences that is high enough to warrant investigation of the nature of these experiences and the meanings that they have for the people having them.

We postulate that there may be cognitive interpretations of experiences of living with ALS that may increase the likelihood of experiencing resentment and hatred. If so, then clinicians could work with ALS patients to learn to interpret events in ways that are less emotionally and physiologically distressing. This is what was personally observed by Oster, first author of this paper and an 18 year survivor of ALS, in his self-case study. Oster has experienced much more positive experiences of living with ALS through working with a psychoanalyst psychologist for over 12 years, and experiences less hate and resentment toward ALS. Oster states that he has learned to see the world differently, based to a large extent upon learning to interpret and understand living with ALS differently (unpublished data). There are no or very few studies about psychological interventions in ALS, as recently pointed noted (Pagnini et al., 2012b). However, psychological treatments with people with other chronic illnesses presented a high capacity to improve the well-being and to reduce the anger response, promoting acceptance (Telford et al., 2006).

Other researchers have evaluated that patient hopefulness is the result of an active style of coping, although their construct of hope was more comprehensive than our definition (Fanos et al., 2008). Furthermore, previous research has demonstrated that lower levels of well-being are associated with reduced longevity (Pagnini et al., 2012a); therefore, we believe that it would be worthwhile for additional research on the relationship between the variables of hate, resentment, and hope in relationship to well-being to help understand whether these variables factor into longevity with ALS.

The analyses of the relationships among resentment, hate, and hope provide interesting results. Subjects who reported hope of living 10 years or more reported less hate and resentment toward ALS. Further research is required to understand hope in relationship to hate and resentment, and to answer the following questions. Is hope in ALS protective regarding the experience of hating and resenting ALS? And/or, do tendencies to experience greater hate and resentment regarding losses, challenges, limitations, and frustrations lessen hope in people with ALS? Aside from the relationship of lower levels of hating ALS being associated with being married and having hope of surviving 10 years post-diagnosis, it is unclear from our findings what causes some people to always hate ALS and others to never hate it. Research is needed to understand the meanings and experiences of the people who always hate ALS and those who report to never hate ALS.

Levels of hate and resentment were correlated; moreover, subjects’ estimation of the level of resentment in the caregiver was related to their own resentment level, without statistical differences between them. To our knowledge, this is a new finding, yet there is the issue that our research relied upon the perception that the person with ALS has of their caregiver. So, we do not know the extent to which this finding reflects actual symmetry between the person with ALS and their caregiver. Researchers have demonstrated symmetry between people with ALS and caregivers in regards to distress, but this has not been previously studied in regards to resentment (Rabkin et al., 2000).

There were differences in gender in the report of resentment and hate related to the illness. Women indicated higher level of resentment and a higher level of hate, with the latter emotion only expressing a tendency toward significance. The novelty of this data suggests caution in its interpretation, but a possible explanation is that women are more devastated by being robbed of their caregiving role as well as by being forced into being a recipient. Cultural norms may play a role in gender differences found. Cultural norms emphasize the physical appearance of the woman being linked to feeling valued, loved, included, but the perception of the body after the diagnosis of ALS may be related to discard, pity, and contempt. Furthermore, men may have a tendency to have been recipients of nurturing and caregiving from not only mothers but also wives, therefore adapting more easily to being put in the role of being taken care of. Further investigations and data are required regarding these cautious interpretations, as well as regarding whether or not some of women’s greater resentment than men about ALS is related to some extent to greater distress with receiving the very personal assistance required with urination and bathing as a recipient of caregiving.

The time since diagnosis is another issue that discriminates hate and resentment levels, as well as reported hope. In our sample, subjects who have had ALS for a longer time reported higher levels of hope as well as lower levels of both hate and resentment. A possible explanation to these findings may be related to the relationship established with the illness and a grief elaboration process. Living with the illness could promote its acceptance; therefore, the amount of time spent with ALS may play a positive role in diminishing hate and resentment toward the illness. Moreover, the reason why people who survive ALS longer have more hope of living longer could be related to the course of the illness being less quick than initially perceived, resulting in less hate and resentment toward ALS.

In the light of our findings, and because of the quality of life issues involved, we suggest that clinicians attempt to understand the meanings of patients’ hate and resentment toward ALS and not take it at face value. Although, it appears that the passage of time may in many cases increase hope and decrease both hate and resentment, it is possible that some people could get stuck in the grieving process because of the immensity of the losses that may occur with ALS.

The investigation of hope provided interesting results about the motivation toward following medical indications. The subjects who have the hope to live more than 10 years or more reported, as a group, more willingness to follow a strict nutrient-dense diet if scientific research indicated that such a diet increased life expectancy by 1 year, but declared that they would not agree to consume drugs with uncomfortable side effects, even if these would increase life expectancy by 1 year. It seems likely that people who already have the hope to maintain a positive level of quality of life may be more interested in maintaining it, as compared to worsening their quality of life to gain 1 year of survival.

It is noteworthy that some subjects, whether or not they have the hope of 10 years survival, would be willing to sacrifice 1 year of life rather than eating a nutrient-dense diet if it were demonstrated to be healthier for ALS. 18% (14 subjects) of the population in our study would be willing to refrain from a healthier diet, instead of extending their lives by 1 year. Slightly less than 5% (four subjects) of the population in our study would be willing to refrain from a healthier diet, instead of extending their lives by two or more years. To our knowledge, this is the first time that people with ALS have reported and been documented to be willing to sacrifice months off of their lives in order to eat whatever they desire. One subject in our study, whose response was categorized as “other” stated that they would only follow a life extending diet if it tasted good. Others reported that it would depend upon the quality of life that they would have, if they were to practice the hypothetical diet.

Additional research is required to discover what the meanings are for the people with ALS who would cut their lives short, rather than choose to focus on their longevity through life style changes. It appears that there is a cost-benefit analysis going on in terms of time, given that over three times as many subjects (14 of them) would refrain from a nutrient-dense diet that would extend their lives for a single year, as compared to the subjects (only four of them) who would refrain from consuming a nutrient-dense diet that would extend their lives by two or more years. Research is needed to understand whether or not actual self-care dietary behavioral choices are predictive of longevity in a way other than what has been established about people with ALS who maintain weight having a greater longevity (Paganoni et al., 2011). Another area deserving research is whether or not hope in ALS is related to longevity, mediated by self-care dietary behavioral choices, dietary, or otherwise.

Caregivers’ resentment, as perceived by the subjects, is related to subjects’ reports of whether their dietary habits have changed as a result of losses associated with the disease. Perhaps this reflects a sense of guilt in the person with ALS, in relationship to the caregiver to whom they may feel burdensome. Some previous papers have reported on a sense of feeling guilty toward the caregiver as one of the most distressing issues for a person with ALS (Bolmsjo, 2001). It seems possible that if a subject indicates higher caregiver resentment, that the reported perception of caregiver’s resentment could be to some extent a projection of an inner sense of guilt. Another possibility is that the person may accurately perceive the high resentment of the caregiver, and therefore experience some guilt that leads to his/her having a lower enjoyment of life, including dietary habits. Clinically addressing and attenuating caregivers’ resentment could possibly reduce the ALS diagnosed persons’ resentment level, to the extent that part of the latter’s resentment of ALS is related to being dependent upon a caregiver with high resentment. Previous research has demonstrated that caregiver depression is related to the depression experienced by the patient, and therefore, addressing caregiver depression may also reduce patient distress (Pagnini et al., 2010, 2012a).

This study presents some limitations. The use of non-validated instruments, such as the survey questions that we have created, may reduce the validity of the results. Further studies with validated questionnaires about anger, hate, and resentment are warranted. The cross-sectional design does not allow any causal inferences between variables, even if these can be discussed in the interpretation. Moreover, the sample size was small and it is possible that some correlations would be underestimated. Finally, the choice of an Internet-based survey may limit the external validity of the study to people with ALS who have access to Internet, and who can easily communicate with a keyboard, augmentative communication, or with personal assistance. The self-selection of the sample may further reduce the generalizability of results, and there was no premorbid attitudinal assessment to compare to the current attitudes. The only inclusion criteria was a self-reported diagnosis of ALS, and we had no chance to verify this information. However, subjects did not receive any compensation, therefore we consider the chance of subjects’ participation without ALS not probable. Moreover, there is a balance between male and females in our sample, but it seems that ALS is lightly more prevalent in males, suggesting that the sample could be non-completely representative. Therefore, further studies with representative samples, longitudinal and experimental designs are required to confirm and deepen the findings presented in this paper.

## Conclusion

The present study is a first attempt to investigate hate, resentment, and hope in ALS, in relationship to each other and in relationship to the willingness to follow a strict nutrient-dense diet, if it were shown to increase longevity. We conducted an online survey, where people with ALS were directly invited to provide answers. Results indicate that there is a massive presence of hope in our sample, with heterogeneous levels of hate and resentment; the three constructs were interrelated. Subjects with hope of living 10 years or more were more likely to report a willingness to make dietary lifestyle changes if such changes were demonstrated to increase longevity. Resentment and hate tended to be higher in people who have had ALS for less time, and in women. People who have lived with ALS for more time expressed a higher level of hope to live 10 years or more.

The main findings of this research indicate that hate, resentment, and hope could be important issues to investigate in future ALS studies. Their roles in the acceptance process of the illness, and how hate and resentment feelings as well as hopelessness may be clinically treated, are important topics to be explored by ALS researchers and clinicians. In particular, the development of specific psychological interventions, or research about existing psychological treatments applied to people with ALS, seems important to improve patients’ and caregivers’ quality of life (Pagnini et al., 2012b).

## Conflict of Interest Statement

The authors declare that the research was conducted in the absence of any commercial or financial relationships that could be construed as a potential conflict of interest.
